# Soluble epoxide hydrolase inhibition Promotes White Matter Integrity and Long-Term Functional Recovery after chronic hypoperfusion in mice

**DOI:** 10.1038/s41598-017-08227-z

**Published:** 2017-08-10

**Authors:** Yuxue Chen, Hao Tian, Ensheng Yao, Yeye Tian, Huaqiu Zhang, Li Xu, Zhiyuan Yu, Yongkang Fang, Wei Wang, Peng Du, Minjie Xie

**Affiliations:** 1 Department of Neurology, Tongji Hospital, Tongji Medical College, Huazhong University of Science and Technology, Wuhan, 430030 PR China; 2 Department of Neurosurgery, Tongji Hospital, Tongji Medical College, Huazhong University of Science and Technology, Wuhan, 430030 PR China; 3 Key Laboratory of Neurological Diseases of Chinese Ministry of Education, the School of Basic Medicine, Tongji Medical College, Huazhong University of Science and Technology, Wuhan, 430030 PR China; 4 Department of Neurology, Zhongshan Hospital, Fudan University, No. 180 Fenglin Road, Shanghai, 200032 PR China

## Abstract

Chronic cerebral hypoperfusion induced cerebrovascular white matter lesions (WMLs) are closely associated with cognitive impairment and other neurological deficits. The mechanism of demyelination in response to hypoperfusion has not yet been fully clarified. Soluble epoxide hydrolase (sEH) is an endogenous key enzyme in the metabolic conversion and degradation of P450 eicosanoids called epoxyeicosatrienoic acids. Inhibition of sEH has been suggested to represent a prototype “combination therapy” targeting multiple mechanisms of stroke injury with a single agent. However, its role in the pathological process after WMLs has not been clarified. The present study was to investigate the role of a potent sEH inhibitor, 1-trifluoromethoxyphenyl-3-(1-propionylpiperidin-4-yl) urea (TPPU), on multiple elements in white matter of mice brain after chronic hypoperfusion. Adult male C57BL/6 mice were subjected to bilateral carotid artery stenosis (BCAS) to induce WMLs. Administration of TPPU significantly inhibited microglia activation and inflammatory response, increased M2 polarization of microglial cells, enhanced oligodendrogenesis and differentiation of oligodendrocytes, promoted white matter integrity and remyelination following chronic hypoperfusion. Moreover, these cellular changes were translated into a remarkable functional restoration. The results suggest that sEH inhibition could exert multi-target protective effects and alleviate cognitive impairment after chronic hypoperfusion induced WMLs in mice.

## Introduction

Small vessel diseases are mainly lesions located in the subcortical structures such as lacunar infarcts and white matter lesions (WMLs), which constitute one of the major causes of vascular cognitive impairment (VCI)^1, 2^. White matter is mainly composed of axonal fibers, oligodendrocytes (OLs), and other glial cells, which is particularly susceptible to ischemic injury^3^. The pathogenic bases of WMLs include hypoperfusion, oxidative stress and inflammation, which in turn lead to endothelial damage, blood-brain barrier (BBB) breakdown, activation of innate immunity and disruption of trophic coupling between vessels and brain cells^4^. Multiple players are involved in the highly complex pathophysiologic responses after stroke. Therefore, therapeutic approaches that target multiple elements of the damage cascade hold considerable promise for the treatment of stroke^5, 6^.

Epoxyeicosatrienoic acids (EETs), which are synthesized by cytochrome P450 (CYP) epoxygenases from arachidonic acid, have potent angiogenic, anti-inflammatory, anti-apoptotic, anti-thrombotic, and anti-oxidant effects^7, 8^. In the brain, EETs exert cytoprotective effects upon several individual components of the neurovascular unit under simulated ischemic conditions *in vitro* ^9–11^. Once formed, EETs are rapidly converted by soluble epoxide hydrolase (sEH), to their corresponding less potent diols known as dihydroxyeicosatrienoic acids (DHETs)^12^. Inhibition of sEH has been demonstrated to have neuroprotective effects against experimental ischemic brain injury^13–16^. Meanwhile, sEH inhibition has been suggested to represent a prototype “combination therapy” targeting multiple mechanisms of stroke injury with a single agent^17^. However, the roles of sEH inhibition in the pathophysiology of chronic cerebral hypoperfusion induced WMLs remain to be determined.

In the present study, we characterized the distribution of sEH expression in the white matter of mice brain. Moreover, the neuroprotective effects of 1-trifluoromethoxyphenyl-3-(1- propionylpiperidin-4-yl) urea (TPPU), a novel potent sEH inhibitor which can cross the BBB^18, 19^, on white matter integrity and functional recovery after chronic hypoperfusion in mice were investigated. Our results showed that TPPU could exert multi-target protective effects and alleviate cognitive impairment after chronic hypoperfusion.

## Results

### Demyelination, axonal loss and cognitive function impairment after BCAS

**Figure 1:**
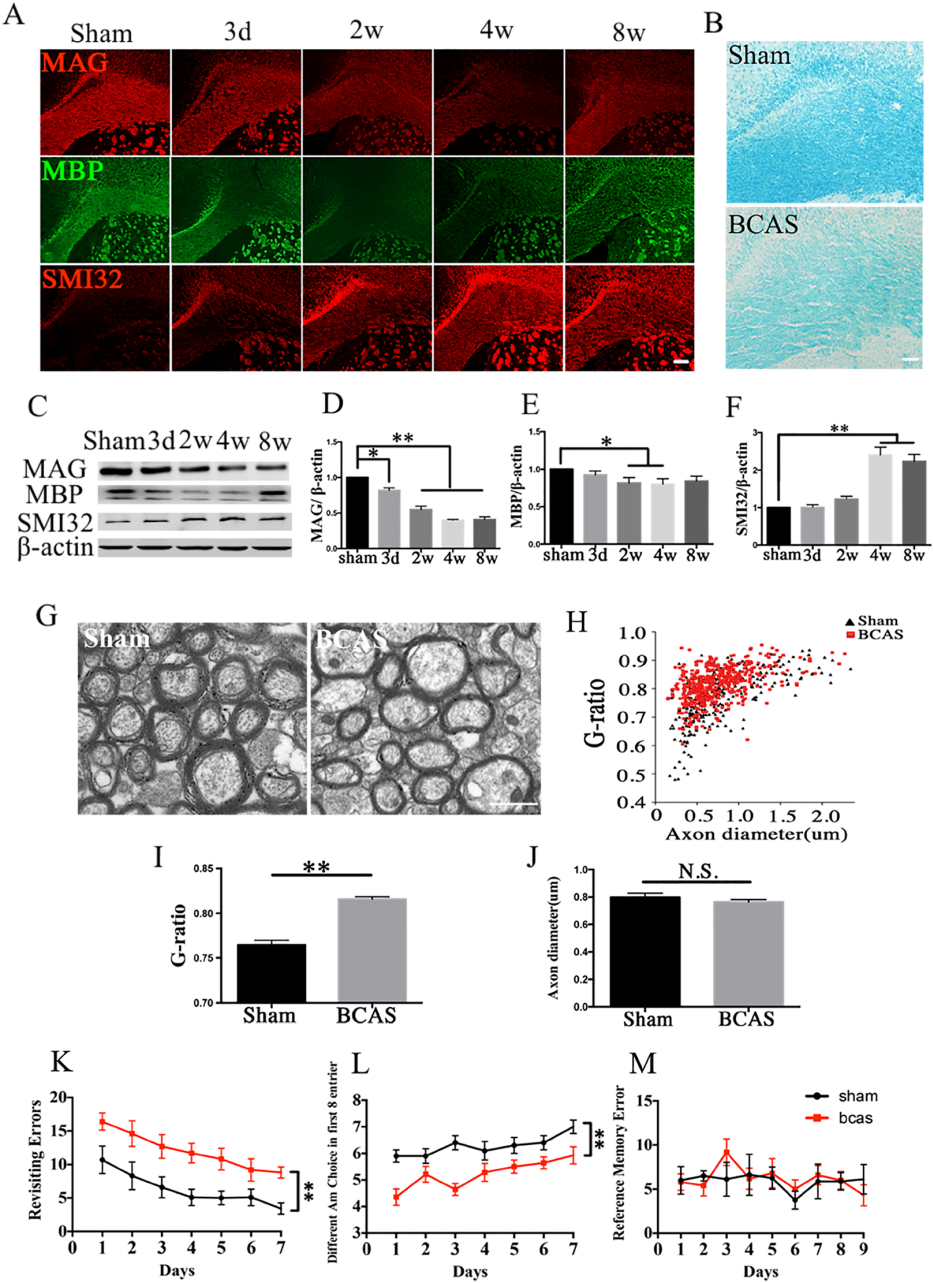
Demyelination, axonal loss and cognitive function impairment after bilateral carotid artery stenosis (BCAS). (**A**) Representative immunofluorescent staining of myelin associated glycoprotein (MAG, red), myelin basic protein (MBP, green), and non-phosphorylated neurofilaments (SMI32, red) in the corpus callosum (CC) of mice in the sham and BCAS groups from days 3 to weeks 8 after operation, n = 4/group. Scale bar represents 100 μm. (**B**) Representative LFB staining in the CC of sham and BCAS groups. LFB staining in the sham group exhibited labeling intensity and characteristic of normal myelination, whereas it was lighter and there were many vacuoles in the BCAS group at 4 weeks after operation, n = 8/group. Scale bar represents 50 μm. (**C–F)** Representative images and statistical analysis of western blots of MAG, MBP, SMI32 and β-actin expression in the CC of sham and BCAS mice at 4 weeks after operation, n = 4/group. (**G**) Representative electron micrographs of white matter in the CC from sham and BCAS animals at 4 weeks after operation. Scale bar represents 1 μm. (**H–J**) Statistical analysis of axon diameter/fiber diameter (G-ratio) and fiber diameter in the sham and BCAS groups, n = 4/group. (**K–M)** The eight-arm radial maze test (Revisiting errors, Different arm choices in the first 8 entries, and Reference errors) was performed to measure cognitive deficits at 4 weeks after BCAS operation, n = 10/group. Values are expressed as mean ± SEM. *P < 0.05, **P < 0.01 vs sham group.

We examined the white matter integrity damage in the corpus callosum (CC) using immunofluorescent labeling for MAG, MBP, and SMI32, respectively. The loss of MBP and MAG immunoreactivity were detected while the abnormal increase of SMI32 expression was observed after BCAS (Fig. 1A). Western blot analysis confirmed the protein expression changes as revealed by immunofluorescent staining (Fig. 1C–F). Next we used LFB staining to evaluate the degree of white matter demyelination at 4 weeks after BCAS. Compared with sham control, the myelinated fibers were disorganized, and vacuoles were frequently observed in the neuropil (Fig. 1B). With respect to their distribution, WM lesions were the most intense in the middle part of corpus callosum (CCm) (1.38 ± 0.74 vs 0 ± 0, *P* < 0.01); were moderate in the paramedian part of the corpus callosum (CCp) (1.00 ± 0.76 vs 0 ± 0, *P* < 0.01), and fiber bundles of the caudoputamen (CPu) (0.88 ± 0.64 vs 0 ± 0, *P* < 0.01); were less severe in the anterior commissure (AC) (0.50 ± 0.76 vs 0 ± 0, *P* < 0.05), and the internal capsule (IC) (0.63 ± 0.74 vs 0 ± 0, *P* < 0.05) in BCAS group compared with sham-operated mice at 4 weeks after BCAS. To investigate whether chronic hypoperfusion could affect the myelin thickness and neuronal fiber integrity, an ultra-structural electron microscopy was carried out. The BCAS mice exhibited significant ultrastructural alterations in myelin and axons, such as collapsed myelin layers, reduced thickness of the myelin sheath, and absence of myelin lamellar pattern (Fig. 1G). A key measure of white matter health is the G-ratio, which showed a significant increase in the CC at 4 weeks after hypoperfusion whereas the axonal diameter showed no significant changes (Fig. 1H–J). The results revealed that there exist demyelination and reduction of myelin thickness in mice after BCAS injury.

Then, we used an eight-arm radial maze to investigate neurological functions at 4 weeks after BCAS. BCAS mice performed much worse in working memory task compared with sham-operated group (Fig. 1K,L; *P* < 0.05). Whereas, no significant difference was showed between the two groups in the reference memory test (Fig. 1M; *P* > 0.05).

### Expression of sEH in white matter of normal and hypoperfusion injured brain

**Figure 2:**
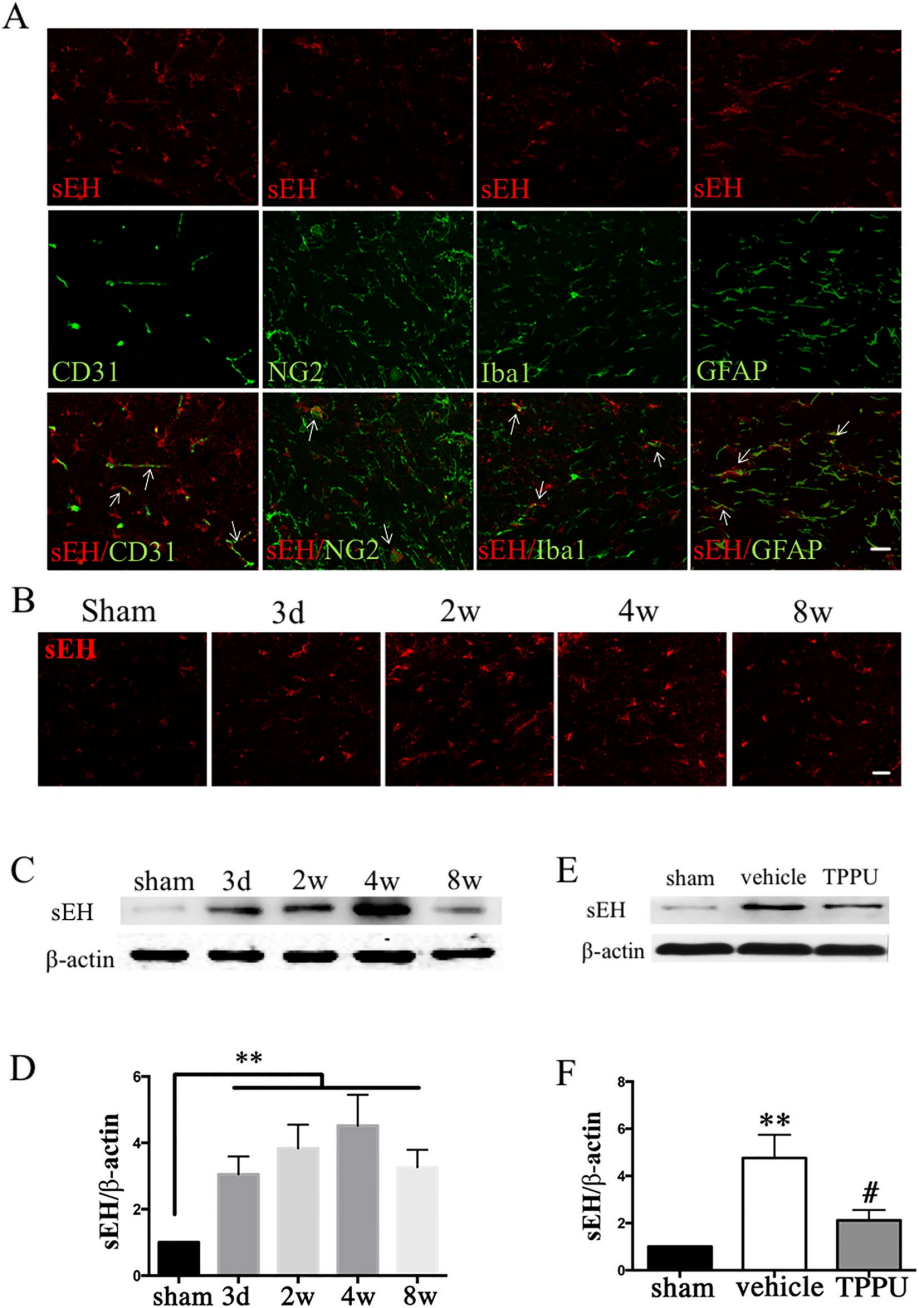
Expression of soluble epoxide hydrolase (sEH) in white matter of normal and hypoperfusion injured brain. (**A**) Double immunofluorescent staining of sEH (red), CD31/NG2/Iba1/GFAP (green), and merge of CD31/NG2/Iba1/GFAP and sEH in the corpus callosum (CC) section. Scale bar represents 20 μm. (**B**) Immunofluorescence of sEH in the CC of mice in sham and bilateral carotid artery stenosis (BCAS) group from days 3 to weeks 8 after operation. Scale bar represents 20 μm. (**C**) Representative western blots of sEH and β-actin expression in the CC of sham and BCAS mice at 4 weeks after operation. (**D**) Statistical analysis of western blots of sEH in the CC of sham and BCAS mice, n = 5/group. (**E**) Representative western blots of sEH and β-actin expression in the CC of sham, vehicle and TPPU treatment groups at 4 weeks after operation. (**F**) Statistical analysis of western blots of sEH in the CC of sham, vehicle, and TPPU treatment groups at 4 weeks after operation, n = 5/group. Values are expressed as mean ± SEM. *P < 0.05, **P < 0.01, vs sham group. ^#^ *P* < 0.05, ^##^ *P* < 0.01, vs vehicle group.

Under normal condition, we found wide distribution of sEH in the CC area of mice brain. Double immunofluorescence staining demonstrated that sEH immunoreactivity co-localized with CD31 positive vascular endothelium, GFAP positive astrocytes and NG2 positive oligodendrocyte progenitor cells (OPCs) in the CC area. Meanwhile, a part of Iba1-positive microglia also showed sEH - immunoreactivity (Fig. 2A).

Subsequently, we investigated the protein expression changes of sEH after BCAS. Immunofluorescence staining revealed that the sEH immunoreactivity was relatively low in sham-operated mice. After BCAS injury, the sEH immunoreactivity began to increase from 3 days following hypoperfusion (Fig. 2B). Consistent with the immunofluorescence staining result, western blot analysis showed that the expression of sEH increased significantly on day 3, remarkably elevated at week 2, peaked on week 4, minor decreased on week 8, but still remained higher compared with the sham-operated group (Fig. 2C,D; *P* < 0.05). After administration with TPPU, the sEH expression was decreased significantly at 4 weeks after operation compared with vehicle control (Fig. 2E,F; *P* < 0.05).

### TPPU attenuates BCAS-induced white matter damage and promotes functional recovery

In our preliminary study, we first investigated the effects of TPPU with various concentrations (0.3, 1.0, or 3.0 mg/kg) at 4 weeks after operation. The results showed that TPPU at each concentration tested could attenuate the white matter injury at different degrees. However, TPPU (0.3 mg/kg) administration induced the most remarkable protective effects (see Supplementary Fig. S1). Therefore, we chose 0.3 mg/kg TPPU concentration in the following experiments.

**Figure 3:**
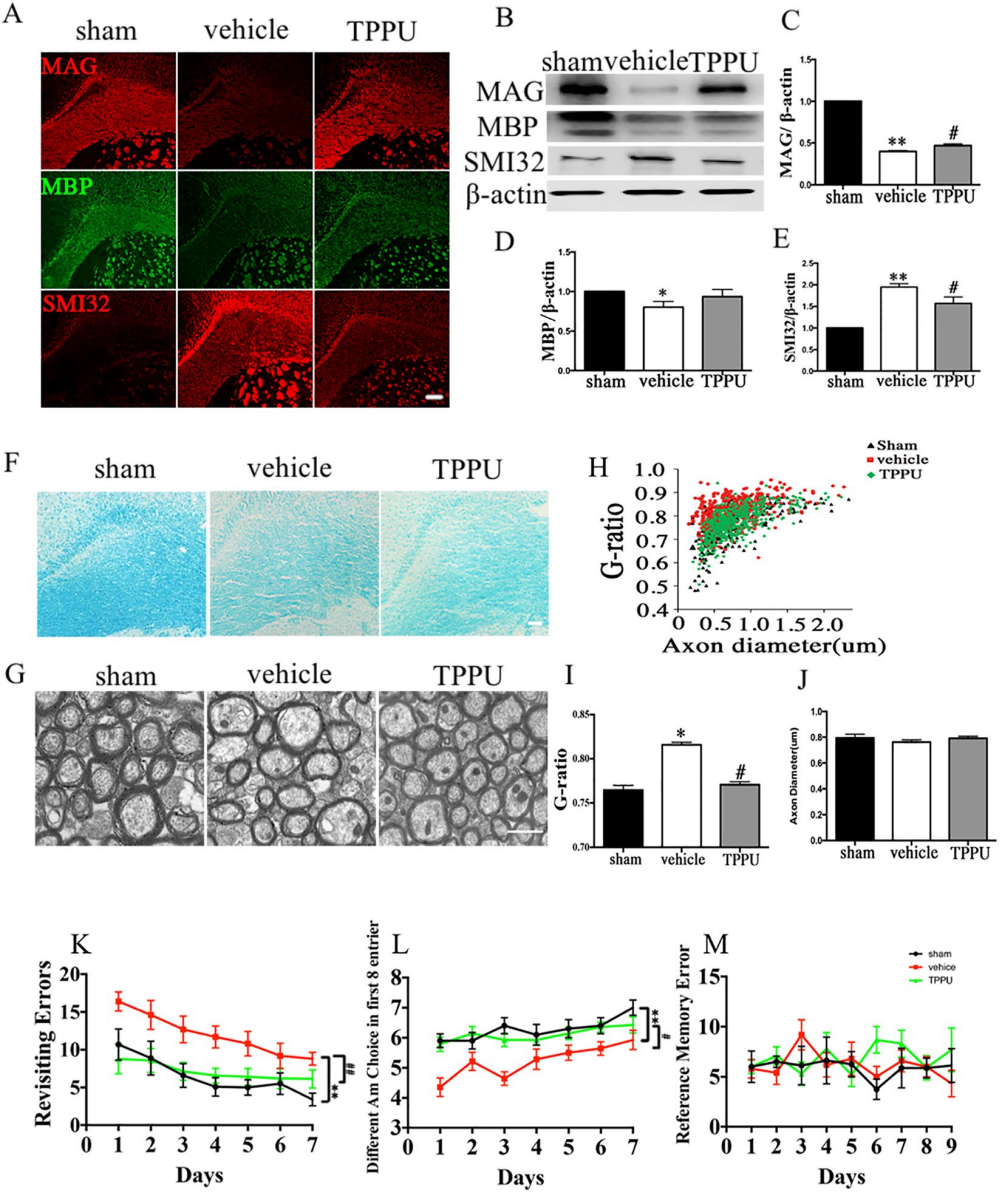
TPPU attenuates bilateral carotid artery stenosis (BCAS) induced white matter damage and promotes functional recovery. (**A**) Representative immunofluorescent staining of myelin associated glycoprotein (MAG, red), myelin basic protein (MBP, green), and non-phosphorylated neurofilaments (SMI32, red) in the corpus callosum (CC) of mice in sham, vehicle and TPPU treatment mice at 4 weeks after operation, n = 5/group. Scale bar represents 100 μm. (**B–E**) Representative and statistical analysis of western blots of MAG, MBP, SMI32, and β-actin expression in the CC of sham, vehicle and TPPU treatment groups 4 weeks after operation, n = 5/group. (**F**) Representative LFB staining in the CC of sham, vehicle and TPPU treatment groups at 4 weeks after operation, n = 5/group. Scale bar represents 50 μm. (**G**) Representative electron micrographs of white matter in the CC from sham, vehicle and TPPU treatment groups at 4 weeks after operation, n = 4/group. Scale bar represents 1 μm. (**H–J**) Statistical analysis of axon diameter/fiber diameter (G-ratio) and fiber diameter in the sham, vehicle and TPPU treatment groups at 4 weeks after operation. (**K–M**) The eight-arm radial maze test (Revisiting errors, Different arm choices in the first 8 entries, and Reference errors) was performed to measure cognitive deficits at 4 weeks in sham, vehicle and TPPU treatment groups, n = 10/group. Values are expressed as mean ± SEM *P < 0.05, **P < 0.01, vs sham group. ^#^ *P* < 0.05, ^##^ *P* < 0.01, vs vehicle group.

Next, we explored whether TPPU treatment could reverse the white matter degradation after BCAS. Compared with vehicle group, administration of TPPU could improve the maintenance of axon-glia integrity assessed by the increase of MAG expression and alleviate the axonal injury indicated by the decreased SMI32 expression at 4 weeks after operation (Fig. 3A–C, E; Supplementary Fig. S2; *P* < 0.05). However, the expression of MBP was not ameliorated significantly after TPPU treatment in comparison with vehicle group (Fig. 3A, B, D; Supplementary Fig. S2; *P* > 0.05). The results indicated that TPPU treatment could effectively protect axon integrity following prolonged cerebral hypoperfusion.

The protective effect of TPPU on white matter demyelination was firstly determined by LFB staining after 4 weeks of chronic hypoperfusion. As shown in Fig. 3F, the white matter demyelination was most intense in CCm post BCAS injury (1.38 ± 0.74). However, TPPU treatment partially attenuated the white matter demyelination (0.25 ± 0.46) (*P* < 0.05 vs vehicle group). Furthermore, we used an electron microscopy to determine whether TPPU rescued myelination abnormalities caused by chronic cerebral hypoperfusion. The mice challenged with BCAS showed evident reduction of myelin thickness measured by an increase of G-ratio at weeks 4 after BCAS, and these changes were significantly attenuated by TPPU treatment (Fig. 3G–I; *P* < 0.05). However, the axon diameter did not change significantly among sham, vehicle and TPPU treatment groups (Fig. 3J). We further studied whether the beneficial effects of TPPU were translated into enhancing neurological function after chronic cerebral hypoperfusion. Administration of TPPU significantly improved the working memory after 4 weeks of BCAS, as demonstrated by a decreased number of revisiting errors and an increased number of different arm choices in the first eight entries compared with the vehicle group (Fig. 3K, L; *P* < 0.05). However, no significant difference was found in the reference memory task among all groups (Fig. 3M; *P* > 0.05). These findings demonstrated that TPPU ameliorated the impairment of working memory after BCAS.

**Figure 4:**
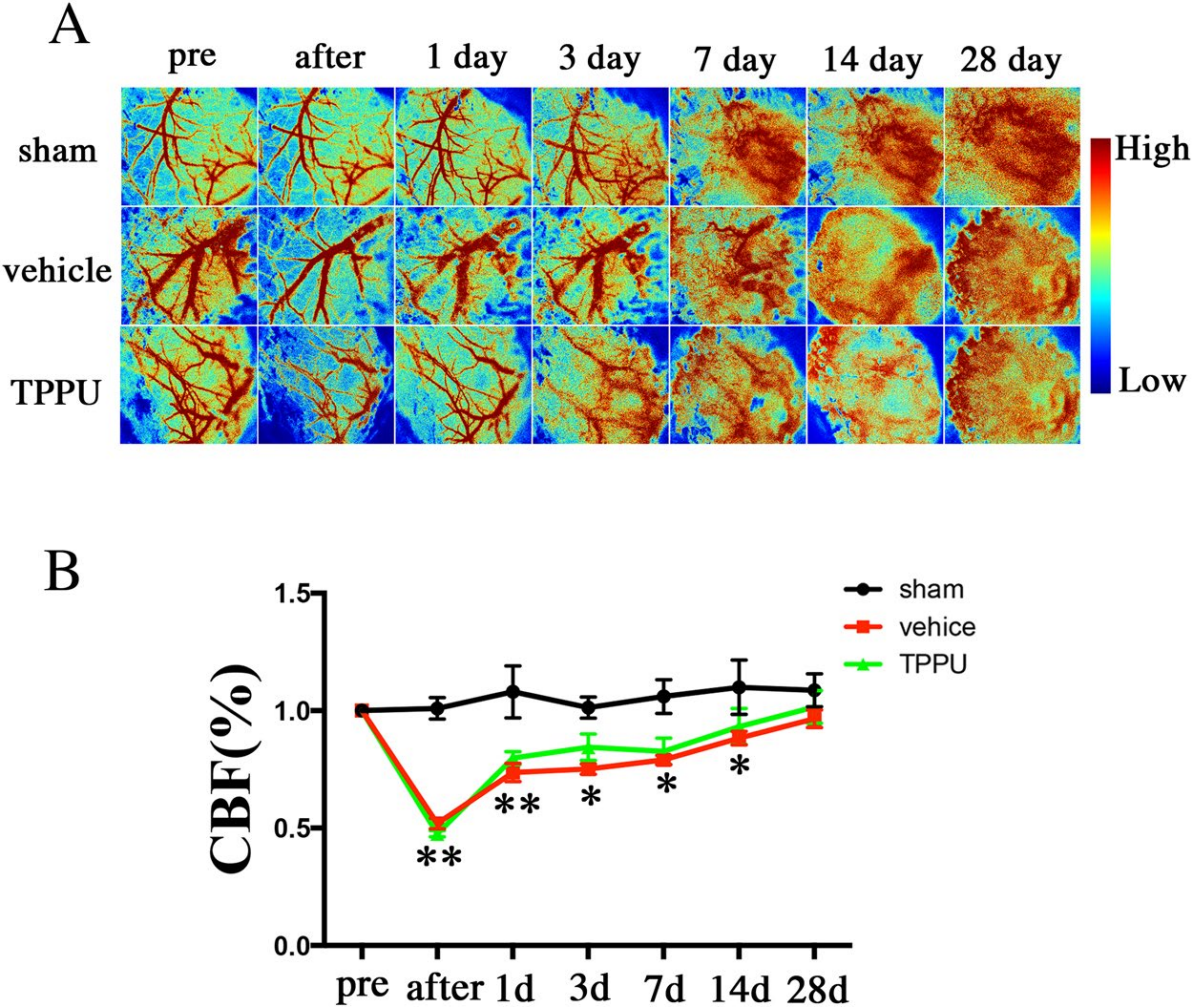
The effects of TPPU on cerebral blood flow (CBF) after BCAS. (**A**) Representative image of CBF measured by laser speckle flowmetry at different time points. (**B**) Statistical analysis of CBF in each group. Values are expressed as mean ± SEM (n = 4/group). **P < 0.01, *P < 0.05, vs sham group.

Meanwhile, we used the laser speckle flowmetry to examine the temporal changes in cerebral blood flow (CBF) (Fig. 4A). Compared with the sham-operated mice, the CBF values (ratio to the preoperative baseline) in the BCAS group decreased significantly after operation (*P* < 0.05). At day 1, the CBF values began to recover but remained significantly lower in comparison to the sham group (*P* < 0.05). However, the CBF values at any time intervals did not change significantly after TPPU treatment compared with vehicle mice (Fig. 4B; *P* > 0.05). Furthermore, we have performed immunofluorescence staining of CD31 to determine the effects of TPPU on microvascular changes in the white matter and found that the number of vascular also showed no significant changes after TPPU treatment (see Supplementary Fig. S3).

### TPPU regulates microglia activation, M2 phenotype polarization and inflammatory factors expression

**Figure 5:**
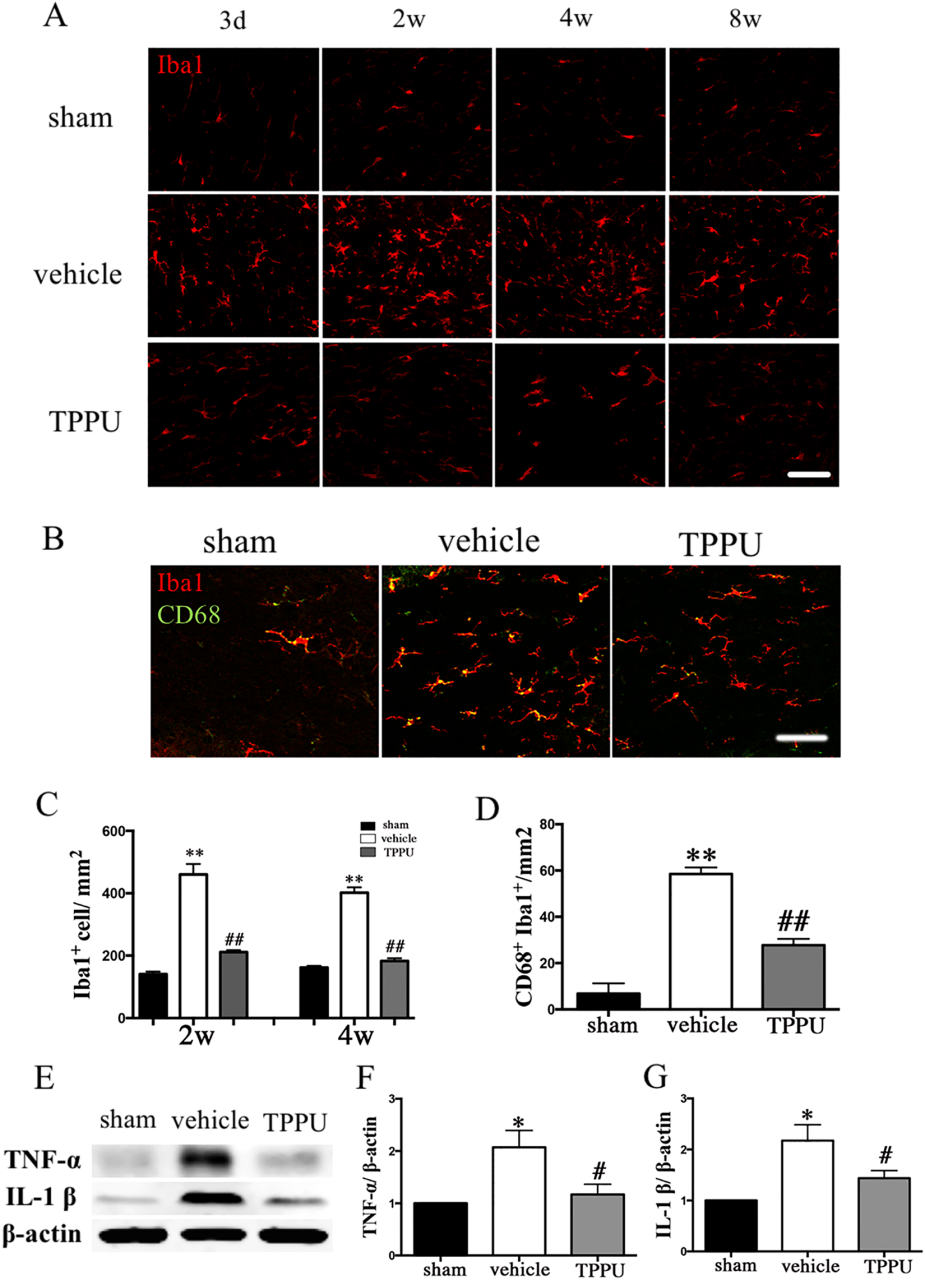
TPPU reduces the microglia activation and inflammatory response after bilateral carotid artery stenosis (BCAS). (**A**) Representative immunofluorescent staining of Iba1 (red) in the corpus callosum (CC) of mice in sham, vehicle and TPPU treatment groups from days 3 to weeks 8 after operation. Scale bar represents 50 μm. (**B**) Representative immunofluorescent staining of Iba1 (red) and CD68 (green) in the corpus callosum (CC) of mice in sham, vehicle and TPPU treatment groups at 4 weeks after operation. Scale bar represents 50 μm. (**C**) Statistical analysis of Iba1 labeled microglia in the CC areas at weeks 2 and weeks 4 were expressed as cells/mm^2^, n = 5/group. (**D**) Statistical analysis of Iba1/CD68 labeled microglia in the CC areas at weeks 4 were expressed as cells/mm^2^, n = 5/group. (**E**) Representative western blots of TNF-α, IL-1β, and β-actin expression in the CC of sham, vehicle and TPPU treatment groups at 4 weeks after operation. (**F–G**) Statistical analysis of western blots signals of TNF-α and IL-1β in the CC of sham, vehicle and TPPU treatment groups, n = 5/group. Values are expressed as mean ± SEM. *P < 0.05, **P < 0.01, vs sham group. ^#^ *P* < 0.05, ^##^ *P* < 0.01, vs vehicle group.

Microglial activation-mediated inflammatory responses may exacerbate ischemic injury. We investigated the effect of TPPU on microglia activation and inflammatory factors expression. Increased number of activated microglia was observed with hypertrophy of cell body as well as thick branches from 3 days after hypoperfusion in the CC. Administration with TPPU remarkably decreased the number of Iba1-positive and CD68/Iba1 positive activated microglia compared with the vehicle groups (Fig. 5A–D; *P* < 0.05).

Pro-inflammatory cytokines including TNF-α and IL-1β contribute to the impaired remyelination in models of demyelination^20, 21^. Western blot analysis showed that BCAS induced a significant up-regulation of TNF-α and IL-1β proteins in the CC at 4 weeks after BCAS injury, which were significantly attenuated by TPPU treatment (Fig. 5E–G; *P* < 0.05).

**Figure 6:**
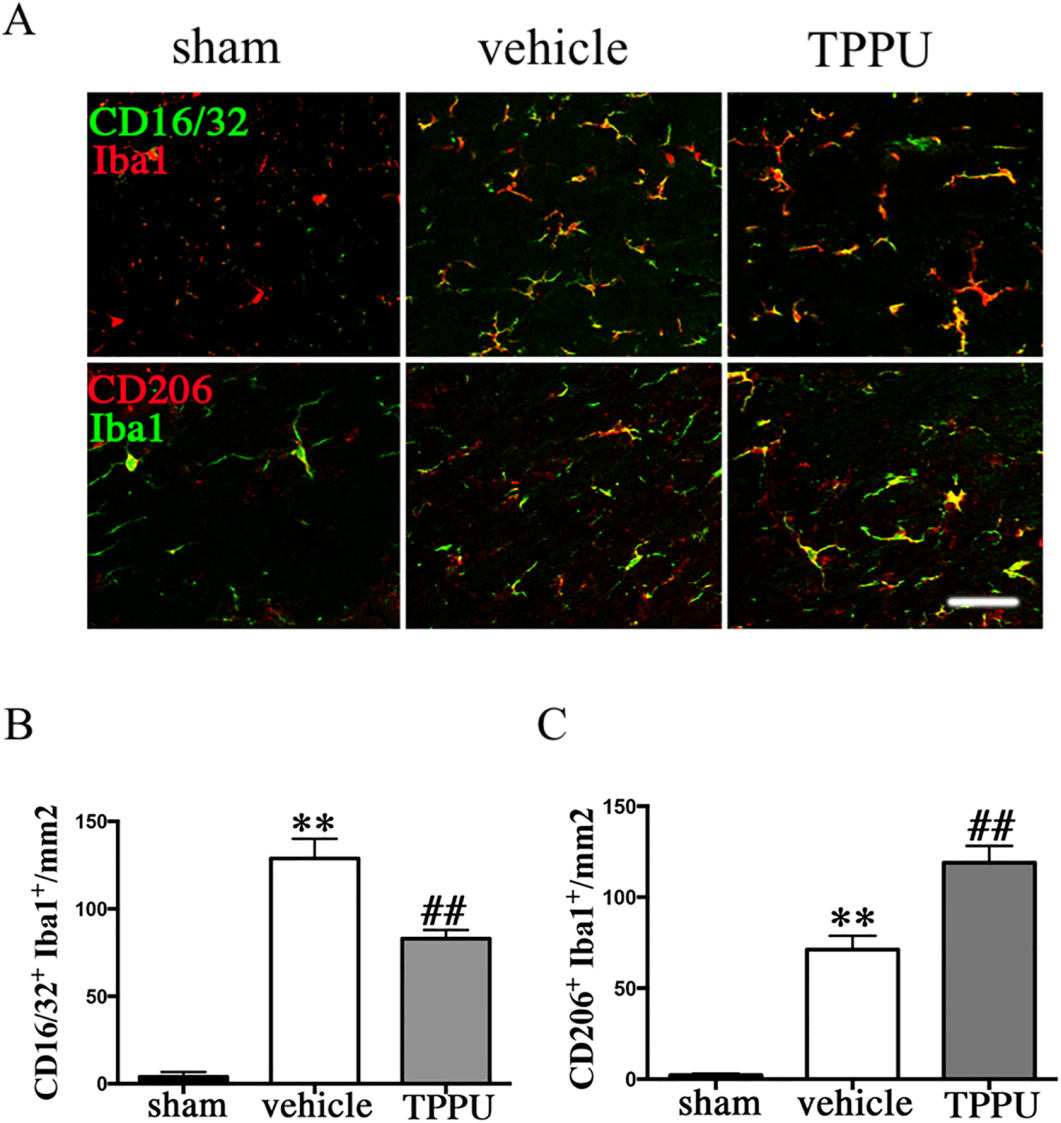
TPPU regulates M2 phenotype polarization occurred in microglia after bilateral carotid artery stenosis (BCAS). (**A**) M1 type microglia in the corpus callosum (CC) at 4 weeks was detected by double staining of CD16/32 (green) and Iba1 (red) in sham, vehicle and TPPU treatment groups. M2 phenotype was detected by double staining of CD206 (red) and Iba1 (green) in sham, vehicle and TPPU treatment groups at 4 weeks. Scale bar represents 50 μm. (**B**,**C**) Statistical analysis of immunofluorescent staining of CD16/32/Iba1 and CD206/Iba1 in the CC of sham, vehicle and TPPU treatment groups at 4 weeks, expressed as cells/mm^2^, n = 5/group. Values are expressed as mean ± SEM. *P < 0.05, **P < 0.01, vs sham group. ^#^ *P* < 0.05, ^##^ *P* < 0.01, vs vehicle group.

Microglia are highly plastic cells that can assume different phenotypes in response to microenvironmental signals. M2 type microglia polarization is essential for efficient remyelination, which may present a therapeutic target of ischemic stroke^22^. To investigate whether TPPU would affect the microglia phenotype within the CC after BCAS, representative M1-associated marker CD16/32 or M2-associated marker CD206 were analyzed by double staining with the microglia/macrophage marker Iba1 at 4 weeks after BCAS (Fig. 6A). The number of both M1 and M2 type microglia increased after BCAS compared with sham control. TPPU inhibited the number of M1 type microglia but further promoted the increase of M2 type microglia compared with vehicle control (Fig. 6A–C; *P* < 0.05).

### TPPU enhances oligodendrogenesis and differentiation of OLs following chronic cerebral hypoperfusion

**Figure 7:**
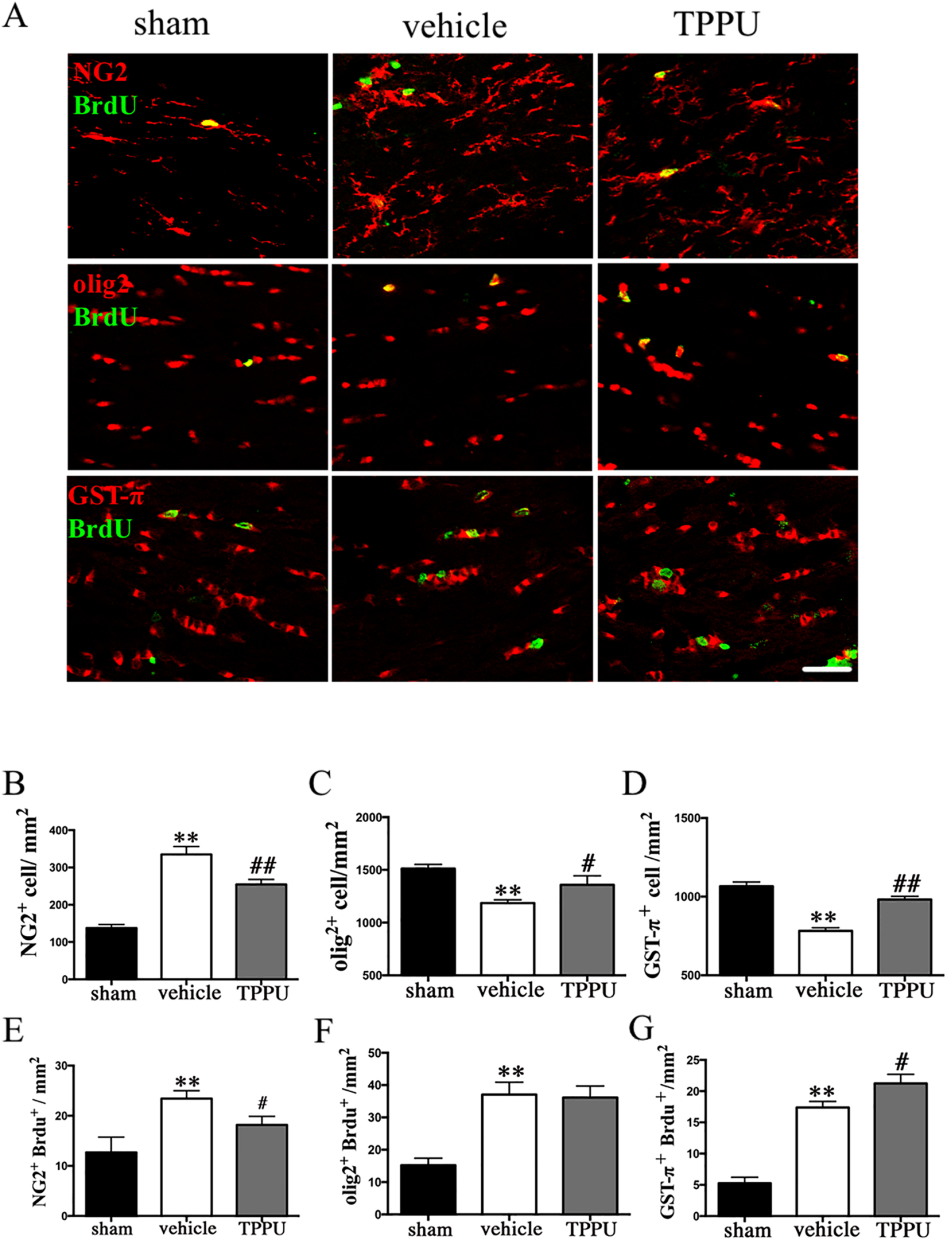
TPPU enhances Oligodendrogenesis and promotes differentiation of oligodendrocytes after bilateral carotid artery stenosis (BCAS). (**A**) Representative image showing the colocalization of bromodeoxyuridine (BrdU; green) and NG2/olig2/GST-π (red) staining in the corpus callosum (CC) of mice in sham, vehicle and TPPU treatment groups at 4 weeks after operation. Scale bar represents 50 μm. (**B–D)** Numbers of NG2 labeled oligodendrocyte progenitor cells (OPCs), olig2 labeled the oligodendrocyte (OL) lineage cells and GST-π labeled mature OLs in the CC were expressed as cells/mm^2^, n = 5/group. (**E–G**) Numbers of BrdU/NG2-dual labeled OPCs, BrdU/olig2-dual labeled the OL lineage cells, and BrdU/ GST-π labeled mature OLs in CC areas were expressed cells/mm^2^, n = 5/group. Values are expressed as mean ± SEM. *P < 0.05, **P < 0.01, vs sham group. ^#^ *P* < 0.05, ^##^ *P* < 0.01, vs vehicle group.

Regeneration of mature myelinating OLs is essential for remyelination and functional recovery after cerebral ischemia. TPPU treatment contributed to oligodendrocyte lineage development after BCAS, thereby facilitating white matter restoration (see Supplementary Fig. S4). Staining of the OPC marker NG2 indicated a progressively increase of positive cells at 4 weeks after BCAS, which was partially reversed by TPPU (Fig. 7A,B; *P* < 0.05). Coincidence with that increase in NG2-positive cells, the numbers of GST-π-positive mature OLs and olig2-positive total oligodendrocyte lineage cells were dramatically reduced. Administration of TPPU attenuated the reduction of GST-π-positive mature OLs and olig2-positive cells (Fig. 7A, C, D; *P* < 0.05).

To determine whether TPPU treatment could facilitate oligodendrogenesis proliferation in chronic cerebral hypoperfusion, BrdU incorporation assay was performed. As shown in Fig. 7A, ischemia stimulated oligodendrogenesis spontaneous proliferation in the CC, as reflected by increased numbers of BrdU-incorporated cells in vehicle-treated group compared with the sham control group. TPPU treatment inhibited the increase of OPCs, as revealed by decreased numbers of BrdU/NG2 double-labeled cells (Fig. 7A, E; *P* < 0.05). Furthermore, TPPU treatment further augmented oligodendrocyte replacement or differentiation, as evidenced by much more colocalization of BrdU/GST-π new mature OLs (Fig. 7A, G; *P* < 0.05). However, TPPU treatment did not change the number of new total OL lineage cells labeled by BrdU/olig2 (Fig. 7A, F; *P* > 0.05). These data indicated that treatment with TPPU enhanced the development of OL-lineage cells by promoting the differentiation of OPCs into mature OLs at 4 weeks after chronic cerebral hypoperfusion.

## Discussion

Approximately one quarter of all strokes in humans occur in white matter^23^, and the progressive nature of white matter lesions (WMLs) often results in severe physical and mental disability. WMLs are characterized by myelin sheath loss and deformation, BBB disruption and glial activation^24^. Traditional studies of WMLs have been focused on the oligodendrocytic death and axonal damage. However, multiple cell types and intercellular signaling cascades contribute to the maintenance of WM integrity and connectivity^25^. Thus, further investigations of WMLs should emphasize cellular networks rather than solely focusing on oligodendrocytes and axons. Combinational protective therapy that targets multiple cellular elements of the oligovascular niche is urgently needed for ameliorating chronic cerebral hypoperfusion induced WMLs^26^. In the present study, we found that sEH inhibition could exert multiple cellular protective effects after chronic cerebral hypoperfusion.

Epoxyeicosatrienoic acids (EETs) are synthesized by cytochrome P450 epoxygenases from arachidonic acid, which have multiple biological effects such as angiogenic, neuroprotection, anti-inflammatory, anti-apoptotic, anti-thrombotic, and anti-oxidant^7, 8, 17^. EETs in the brain have been shown to exert cytoprotective effects in several components of the neurovascular unit under ischemic conditions^9, 27, 28^. However, EETs can be metabolized rapidly by sEH into the corresponding inactive dihydroxyeicosatrienoic acids (DHETs), which possess fewer biological activities. A body of evidence shows that inhibition of sEH is a potential therapeutic target for the treatment of brain ischemia^8, 15, 29, 30^. TPPU is one of the most recently synthesized, stable, BBB permeable potent sEH inhibitors, which has been shown to stabilize EETs *in vivo* ^18, 31–33^. Here we provide the first *in vivo* evidence showing that sEH inhibitor TPPU could promote white matter integrity and remyelination, regulate microglia activation and inflammatory response, and promote oligodendrogenesis and remyelination following chronic cerebral hypoperfusion. Moreover, these cellular changes were translated into a remarkable functional restoration. The data suggest that sEH inhibition is a promising multi-mechanism therapeutic target for the treatment of demyelinating diseases including ischemic WMLs.

Accumulating evidence indicates an active role of microglia in the demyelination/remyelination during ischemic white matter injury^28, 34, 35^. Microglia activation is a prominent feature of demyelinated lesions observed in chronic demyelinating disease of the CNS^36^. Microglia may present diverse phenotypes and play dualistic roles in brain injury and recovery. Classically activated M1 microglia/macrophages are pro-inflammatory and exert detrimental effects on the ischemic brain, whereas alternatively activated M2 microglia/macrophages are anti-inflammatory and protective^37^. M2 polarization of microglia is essential for efficient remyelination in CNS regeneration^38^. Promoted white matter integrity was observed after cerebral ischemia by facilitating microglial polarization toward the beneficial M2 phenotype^38^. EETs signaling has been shown to possess potent anti-inflammatory effects. Pharmacological inhibition or deletion of the gene of sEH decreases inflammatory response to lipopolysaccharide and ischemic insult^39–41^. We previously have shown that administration of sEH inhibitor significantly suppresses microglia activation and alleviates white matter injury after spinal cord injury^28^. Consistent with these studies, we demonstrated that sEH inhibitor TPPU significantly attenuated microglia activation and the expression of pro-inflammatory cytokines. Moreover, administration of TPPU dramatically increased the M2 phenotype of microglia, which could alleviate inflammatory reaction and help to tissue repair and remodeling by clearing up debris and producing anti-inflammatory cytokines and growth factors^22, 42^. The dominant M2 type microglial cells observed in this study might be attributed to the direct effect of the TPPU to microglia as well as the results of milder white matter lesion and less inflammation in the white matter after TPPU treatment. However, microglia may exert their multiple effects on demyelination/remyelination after white matter damage which might not act by solely M1/M2 phenotype transition. Owing to the dual-faced role of microglia, our results suggest that TPPU induced microglial reactivity changes which may create an favorable environment for remyelination in white matter after chronic cerebral hypoperfusion.

As the myelin-producing cells in the CNS, OLs and its precursor cells are known to be highly susceptible to ischemic injury^43^. Furthermore, their loss causes demyelination, impairment of axonal conduction, and ultimately axon death, which are pathologic hallmarks of ischemic white-matter disease associated with VCI^44^. Successful regeneration of OLs is essential for remyelination and axonal preservation after brain injuries^45^. However, just a small number of OPCs can develop into mature OLs, resulting in insufficient remyelination and unsuccessful white matter repair^46, 47^. The therapeutic approaches that can stimulate and protect endogenous oligodendrogenesis have been used to promote WM reorganization^48^. Our previous study has showed that sEH inhibition can help to facilitate remyelination in white matter after spinal injury^28^. In this study, the number of mature OLs decreased following chronic cerebral hypoperfusion. Treatment with TPPU promoted the percentage of OLs and OPCs in the OL lineage. We found that the numbers of BrdU/NG2, BrdU/olig2 and BrdU/GST-π cells increased in the CC after chronic cerebral hypoperfusion. TPPU treatment further augmented oligodendrocyte replacement, as evidenced by smaller colocation of BrdU/NG2 OPCs and greater colocalization of BrdU/GST-π new mature OLs. Finally, numbers of newborn cells labeled with the pan-OL lineage marker olig2 and BrdU were not changed by TPPU, suggesting that the observed changes reflected a shift in cell numbers towards a mature state rather than an overall loss of cells in the OL lineage. TPPU treatment enhanced generation of new OLs from progenitor cells, which are critical for the control of the oligodendrogenesis and maturation. These findings suggest a robust process of OLs regeneration and a potential partial reparative response with the treatment of TPPU following chronic cerebral hypoperfusion induced white matter injury.

Meanwhile, EETs are best known for their role as potent vasodilators, including in the cerebral circulation^49, 50^. The effect of sEH inhibition on the improvement of CBF after ischemic injury is controversial^14–16, 51^. In this study, the CBF values measured by using the laser speckle flowmetry at any time intervals did not ameliorate significantly after TPPU treatment compared with vehicle mice. Moreover, the number of vessels showed no significant changes after TPPU treatment. Our results suggest that sEH inhibition might exert multiple targets protective effects rather than its cerebrovascular effects after chronic hypoperfusion induced white matter injuries.

Cognitive dysfunction depends on volume and strategic location of brain infarction, site and range of cerebral WM, and other co-existent pathologies^52^. Decreased WM integrity induced by ischemia has been reported to tempt disturbance of communication between neurons^53^ and contribute to the persistence of long-term neglect and cognitive deficits in stroke^54^. Preserving white matter integrity could promote neurological behavioral functions. In the current study, we found that chronic hypoperfusion induced severe myelin loss, axon-glial integrity and axonal damage. These pathological changes ultimately resulted in meylination and working memory impairment. However, TPPU treatment ameliorated the white matter integrity and remyelination following chronic cerebral hypoperfusion, which promoted the recovery of neurological functions.

In summary, our present data may provide novel insights into the roles of pharmacological inhibition of sEH in a multiple target–protective effects after chronic cerebral hypoperfusion-induced WMLs. Enhanced sEH inhibition in WM might regulate microglia reactivity and inflammatory response, promote proliferation and differentiation of OL lineage cells, and restore white matter integrity, all of which are likely to contribute to long-term functional recovery after chronic cerebral hypoperfusion. Although further studies are required to reveal the down-stream targets and underlying mechanism of sEH inhibition, sEH inhibition may serve as a promising therapeutic target for the treatment of demyelinating diseases such as chronic hypoperfusion induced WMLs.

## Materials and Methods

### Animals and Model Preparation

All the experiments were conducted in accordance with the National Institute of Health Guide for the Care and Use of Laboratory Animals and approved by the Institutional Animal Care and Use Committee at Tongji Medical College, Huazhong University of Science and Technology. Adult male C57BL/6 mice (10–12 weeks old) weighing between 20 and 28 g were randomly assigned into sham-operated (n = 30), vehicle treatment (n = 50), and TPPU treatment (n = 60) groups. Mice were given access to a 12-hour light-dark cycle in a temperature-controlled facility, with free access to food and water.

TPPU, a potent sEH inhibitor, was developed and generously provided by Dr. Bruce Hammock, University of California, Davis, CA. TPPU was first dissolved in dimethyl sulphoxide (DMSO) and then diluted by sterile saline solution to reach the final concentration (the final concentration of DMSO was 1%). In the present study, the vehicle control was 1% DMSO in sterile saline solution.

Bilateral carotid artery stenosis (BCAS) is a simple and reproducible mouse model to induce chronic cerebral hypoperfusion, which has been reported previously^55^. Briefly, mice were under anesthesia with isoflurane (1–2% by face mask) and two 0.18 mm microcoils (Sawane Spring Co, Sawane, Japan) were wrapped around common carotid arteries. The sham-operated mice were only received bilateral exposure of the CCAs. Mice with any neurological deficiency, such as visual field defect and limb hemiplegia would be excluded. The fatality of this study was 5%.

All the animals were randomly and blindly assigned into vehicle or TPPU treatment groups. The TPPU and vehicle treatment groups were randomly intraperitoneally (i.p) with different doses of 0.3, 1, or 3 mg/kg and 1% DMSO as vehicle control starting at 1 day after BCAS and daily until 1 day before sacrificed. The mice were sacrificed under anesthesia on day 3, week 2, 4, and 8 after BCAS.

### Bromodeoxyuridine Injections

To label proliferating cells, mice received daily i.p injection with 5′-bromo-2′-deoxy-uridine (BrdU, 50 mg/ body weight in sterile saline, Sigma Chemical Co., St. Louis, MO, USA) for 2 weeks. The BrdU was injected at 2 weeks after operation, and then mice were sacrificed 2 weeks later.

### Functional Test

To evaluate neurological deficits and recovery after stroke, an eight-arm radial maze test was performed at 4 weeks after BCAS as previously described^56^. Identical food wells were placed at the distal end of each arm. From one week before pre-training, mice were deprived of food until the body weight was reduced to 80–85% of the initial level. All mice were then given 3 daily sessions of pre-training to facilitate habituation to the apparatus. Each mouse was placed in the central starting platform and allowed to explore and consume food pellets. The test was completed when the mouse found food in all eight arms or when 15 minutes had elapsed. The pellets were then placed only in the four food wells for the test. The arms with pellets were called working arms and the others were called reference arms. Working memory errors (WME) occurred when the mice entered the same working arm for the second time. Reference memory errors (RME) occurred when the mice entered the reference arm for the first time. Both WME and RME were recorded. The animals completed one trial per day.

### Immunofluorescence Staining and Quantification

Mice were sacrificed under deep anesthesia at determined time points. The brains were transcardially perfused with ice-cold normal saline, followed by 4% paraformaldehyde (PFA) in 0.01 M phosphate-buffered saline (PBS). Brain tissues were removed immediately after perfusion, post-fixed in 4% PFA overnight at 4 °C and cryopreserved with successive 20% and 30% (w/v) sucrose in 4% PFA for two days. The brain tissues were cut at 12 μm thickness from the anterior aspect of the corpus callosum (bregma 0.26 mm) to the anterior aspect of the hippocampus (bregma 0.94 mm), according to the mouse brain atlas and mounted to poly-L-lysine-coated slides for immunofluorescent staining.

Briefly, the sections were rinsed in PBS, blocked in 10% bovine serum albumin and 0.5% Triton X-100 to bind nonspecific antigen for 1–2 h at room temperature, then incubated with primary antibody for 1–2 days at 4 °C. The following primary antibodies were used: rat anti- MBP (1:100, Millipore, USA), mouse anti-MAG (1:200, Millipore, USA), rabbit anti-SMI32 (1:200; Abcam, USA), goat anti-sEH (1:50; Santa Cruz, USA), rabbit anti-Iba1 (1:200; Wako, Japan), rabbit anti-olig2 (1:200; Abcam, USA), goat anti-CD68 (1:100; R&D, USA), rat anti-CD16/32 (1:100; BD bioscience, USA), mouse anti-CD206 (1:100; Merck Millipore, USA), rabbit anti-NG2 (1:200, Millipore, USA), mouse anti–adenomatous APC (1:200, Millipore, USA), rabbit anti- GST-π (1:200, Medical and Biological Laboratories, Japan), rabbit anti-GFAP (1:200, Millipore, USA), mouse anti-CD31 (1:200; Abcam, USA), and mouse anti-BrdU (1:100; Santa Cruz, USA). After conjugation with the primary antibodies, the sections were rinsed in PBS, incubated with corresponding secondary antibodies purchased from Jackson ImmunoResearch Laboratories Inc. (West Grove, PA, USA) for 1 h at room temperature. For BrdU/NG2, BrdU/GST-π and BrdU/Olig2 double labeling, the sections were incubated in primary antibody followed by 30 min 2 N HCl incubation at 37 °C. Then sections were blocked again and incubated in anti-BrdU primary antibody followed by secondary antibody incubation. Finally, sections were observed blindly under an Olympus BX51 fluorescent microscope (Olympus, Japan) or laser scanning confocal microscope (Olympus, FV500, Japan). Immunopositive cell counts present as the mean number of cells per square millimeter were quantified by a blinded investigator using Image J software (National Institute of Health, Bethesda, MD, USA).

### Luxol Fast Blue (LFB) Staining

LFB staining was used to observe histological changes after BCAS. The samples were post-fixed in 4% PFA and then dehydrated in a graded ethanol series. Sections were deparaffinized and incubated in 0.1% LFB solution (Goodbio Technology Co Ltd, Wuhan, China) at 56 °C for 8–10 hours. Slides were cooled at room temperature and stained sections were differentiated in Li_2_CO_3_ for 5 min, then briefly in 70% ethanol, followed by counterstaining with eosin. Dehydration was performed in graded alcohol baths and xylene. Reaction was performed using four slices per animal and analysis was performed in five panels per reaction. The severity of the WMLs was graded as normal (grade 0), disarrangement of the nerve fibers (grade 1), formation of marked vacuoles (grade 2), or disappearance of myelinated fibers (grade 3) as described previously^55^.

### Electron microscopy

Animals in sham, vehicle and TPPU treatment groups were perfused with 4% PFA/2.5% glutaraldegyde buffered with 0.1 mol/L phosphate buffer, then removed immediately at 4 weeks after BCAS. Corpus callosum tissues were quickly sectioned into 1 mm thick sections and subsequently immersed in tissues in 2.5% glutaraldegyde overnight at 4 °C. The samples were post-fixed in 1% OsO_4_ for 2 hours at 4 °C in the dark, block-stained in 1% uranyl acetate for 1 hour, dehydrated with a graded series of aqueous alcohol solutions, and embedded in epoxy resin. Samples were cut with an EM UC7 ultramicrotome (Leica Microsystems (UK) Ltd, Milton Keynes), stained with uranyl acetate and lead citrate, and examined in a Tecnai G^2^ 20 TWIN transmission electron microscope (FEI UK Ltd., Cambridge, England). The extent of myelination was quantitatively compared by determining G-ratios, which were calculated by dividing the diameter of the axon by the diameter of the entire myelinated fiber^57^. At least 100 axons were measured for each brain. Measurements and image processing was performed using Image J.

### Western blot

Mice were sacrificed at observation times, and the corpus callosum tissues were extracted. Brain tissues were lysed with RIPA lysis buffer containing PMSF and protease inhibitor cocktail (Roche, USA), which were sonicated on ice for 30 min. Following sonication, lysates were centrifuged at 12,000 rpm at 4 °C for 20 min to obtain supernatants. The supernatants were collected for the protein concentration assay using a bicinchoninic acid (BCA) Kit (Beyotime, Shanghai, China). Then, the proteins were mixed with loading buffer (31.2 mM Tris, 1% sodium dodecyl sulfate, 5% glycerol, and 2.5% β-mercaptoethanol) and boiled for 10 min. Protein samples (20 μg) were electrophoresed on 10% SDS-PAGE separated and then transferred to nitrocellulose membrane (0.45 μm, Millipore, USA). Blots were blocked by 5% fat-free dry milk in TBS containing 0.25% Tween 20 for 1 h at room temperature, and incubated at 4 °C overnight with the following primary antibodies: mouse anti-β-actin (1:1000; Santa Cruz, USA), rat anti-MBP (1:500, Millipore, USA), mouse anti-MAG (1:500, Millipore, USA), mouse anti-SMI32 (1:1000, Abcam, USA), goat anti-sEH (1:500; Santa Cruz, USA), rabbit anti-IL-1β (1:500, Santa Cruz, USA), and mouse anti-TNF-α (1:500, Santa Cruz, USA). Thereafter, the membranes were then incubated with the appropriate secondary antibodies for 1 h at room temperature. The resulting digital images were analyzed by Image J to obtain the optical densities (OD) of signals. Protein was normalized relative to β-actin as expressed as a ratio compared to sham mice.

### Cerebral blood flow (CBF) Measurement

Following BCAS, the CBF was measured by laser speckle flowmetry (Omegazone, Omegawave Inc, Tokyo, Japan). Under deep anesthesia with isoflurane (1–2% by face mask), the skin overlying the skull was reflected. The laser speckle flowmetry was used to monitor the CBF at seven time points, i.e., before surgery, after stenosis on both carotid common arteries, 1 day after BCAS, 3 days after BCAS, 1 week after BCAS, 2 weeks after BCAS, and 4 weeks after BCAS. The CBF values were expressed as a percentage of the baseline value. For quantitative analyses, at least four regions of interest (ROIs) in each mouse were analyzed.

### Statistical Analysis

All measurements were performed by researchers blinded to each group and condition. Data are expressed as the mean ± SEM. Significance of difference between two groups was determined by t-test. For multiple comparisons, one-way or two-way analysis of variance (ANOVA) was followed by Tukey’s post hoc test. Data were analyzed using GraphPad Prism 6.0 (GraphPad Software, Inc, La Jolla, CA). Differences were considered significant if *P* < 0.05.

## Electronic supplementary material

Supplementary information
