# Supplementary material for: Soluble epoxide hydrolase inhibition Promotes White Matter Integrity and Long-Term Functional Recovery after chronic hypoperfusion in mice
Source: Sci Rep. 2017 Aug 10;7:7758. doi: 10.1038/s41598-017-08227-z (PMC5552839; doi:10.1038/s41598-017-08227-z)
Supplement: Supplementary file 1 — Supplementary information [file 41598_2017_8227_MOESM1_ESM.pdf]

ONLINE SUPPLEMENT

Soluble epoxide hydrolase inhibition Promotes White Matter Integrity and Long-Term Functional Recovery after chronic hypoperfusion in mice

Yuxue Chen[1], Hao Tian[1], Ensheng Yao[1], Yeye Tian[1], Huaqiu Zhang[2], Li Xu[1,3], Zhiyuan Yu[1,3], Yongkang Fang[1], Wei Wang[1,3], Peng Du[4*], Minjie Xie[1,3*]

Supplemental Figures and Legends

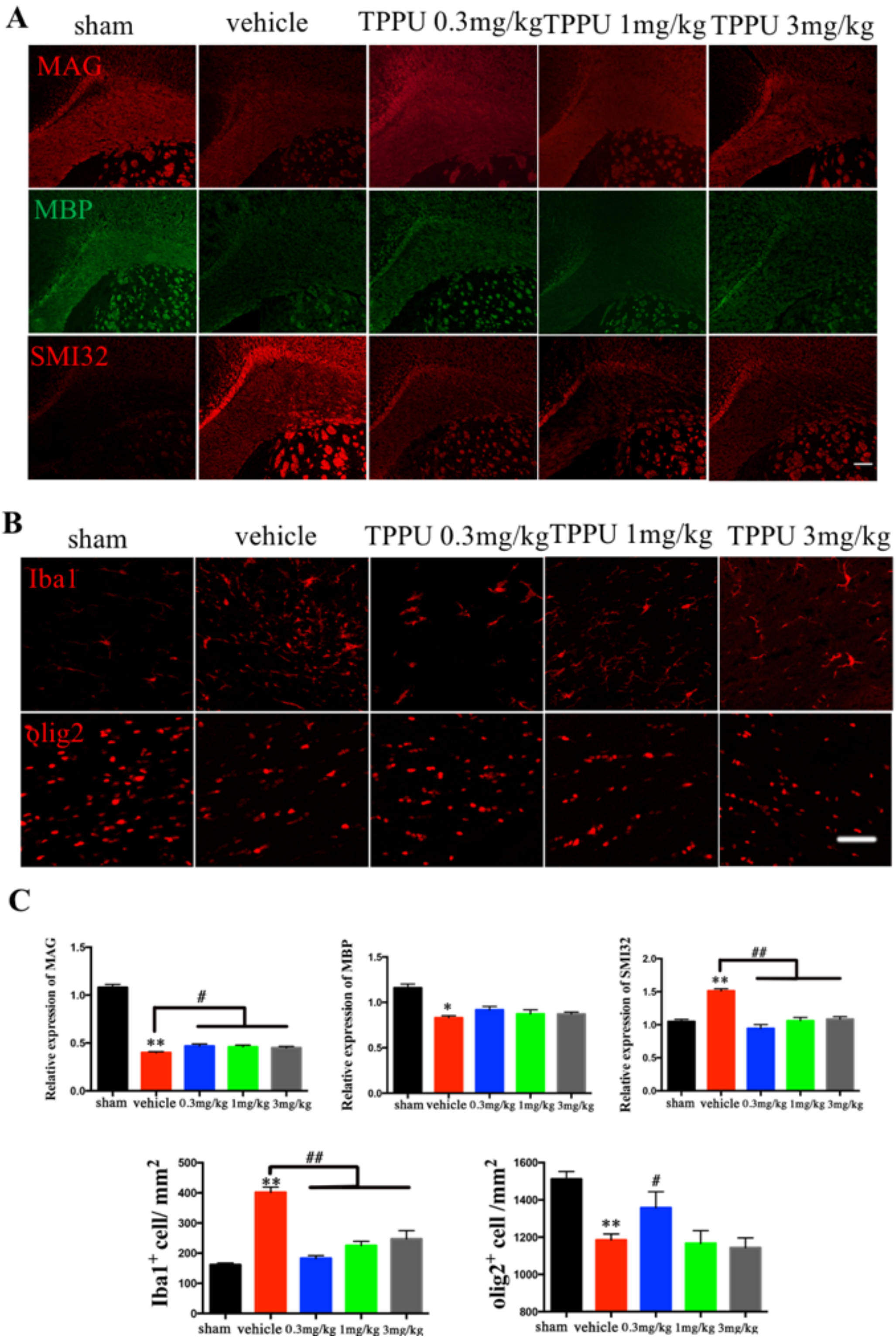

Supplementary Figure S1. Evaluation the effects of different dose of TPPU in mice after bilateral carotid artery stenosis. **(A)** Representative immunofluorescent staining

of myelin associated glycoprotein (MAG, red), myelin basic protein (MBP, green), and phosphorylated neurofilaments (SMI32, red) in the corpus callosum (CC) of mice in the sham, vehicle and different dose of TPPU treatment groups at 4 weeks after operation. Scale bar represents 100 μm. **(B)** Representative immunofluorescent staining of Iba1 (red), olig2 (red) in the CC of mice in the sham, vehicle and different dose of TPPU treatment groups at 4 weeks after operation. Scale bar represents 50 μm. **(C)** Statistical analysis of immunofluorescent staining of MAG, MBP, SMI32, Iba1 and olig2 in the CC of sham vehicle and different dose of TPPU treatment groups at 4 weeks after operation. Values are expressed as mean ± SEM (n=4/group). *P<0.05, **P<0.01, vs sham group. $^{\#}P$<0.05, $^{\#\#}P$<0.01, vs vehicle group.

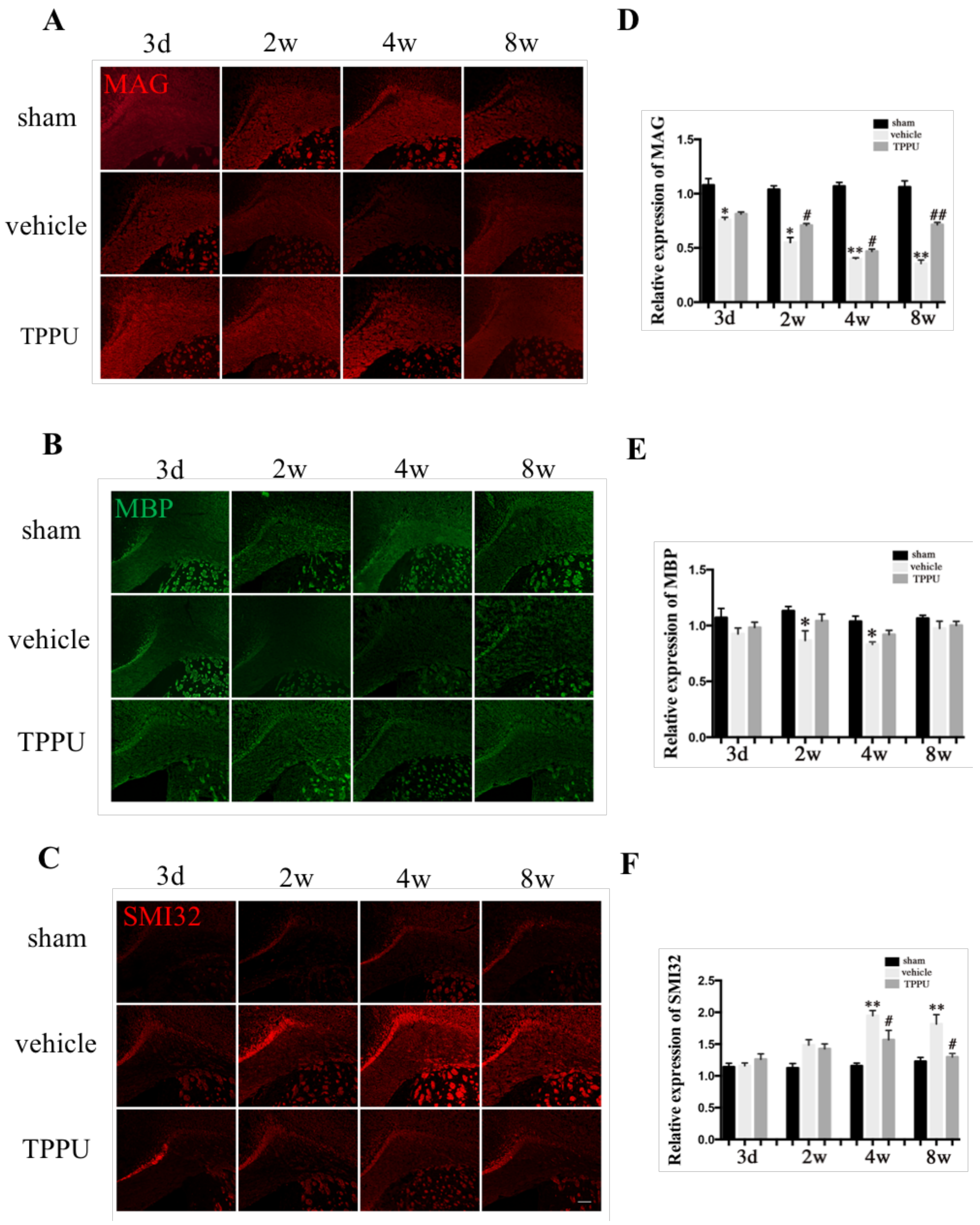

Supplementary Figure S2. TPPU promotes white matter integrity at different time points after bilateral carotid artery stenosis. **(A-C)** Representative immunofluorescent staining of myelin associated glycoprotein (MAG, red), myelin basic protein (MBP, green), and phosphorylated neurofilaments (SMI32, red) in the corpus callosum (CC) of mice in sham, vehicle and TPPU treatment groups from days 3 to weeks 8 after

operation. Scale bar represents 100 μm. (**D-F**) Statistical analysis of immunofluorescent staining of MAG, MBP, and SMI32 in the CC of sham, vehicle and TPPU treatment groups from days 3 to weeks 8 after operation. Values are expressed as mean ± SEM (n=5/group). *P<0.05, **P<0.01, vs sham group. $^{\#}P$<0.05, $^{\#\#}P$<0.01, vs vehicle group.

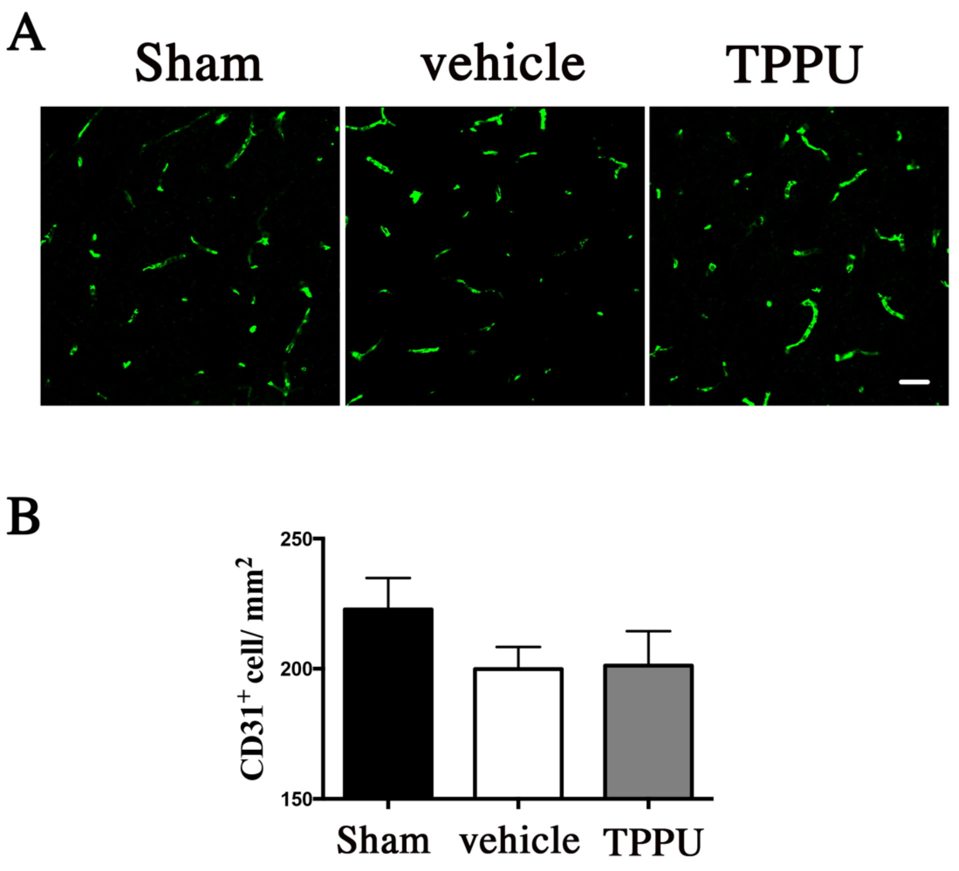

Supplementary Figure S3. The effects of TPPU on microvascular changes at 4 weeks after BCAS. **(A)** Representative immunofluorescent staining of CD31 in the corpus callosum (CC) of mice in sham, vehicle and TPPU treatment groups at 4 weeks after operation. Scale bar represents 20 μm. **(B)** Statistical analysis of immunofluorescent staining of CD31 in the CC of sham, vehicle and TPPU treatment groups, expressed as cells/mm^2. Values are expressed as mean ± SEM (n=4/group).

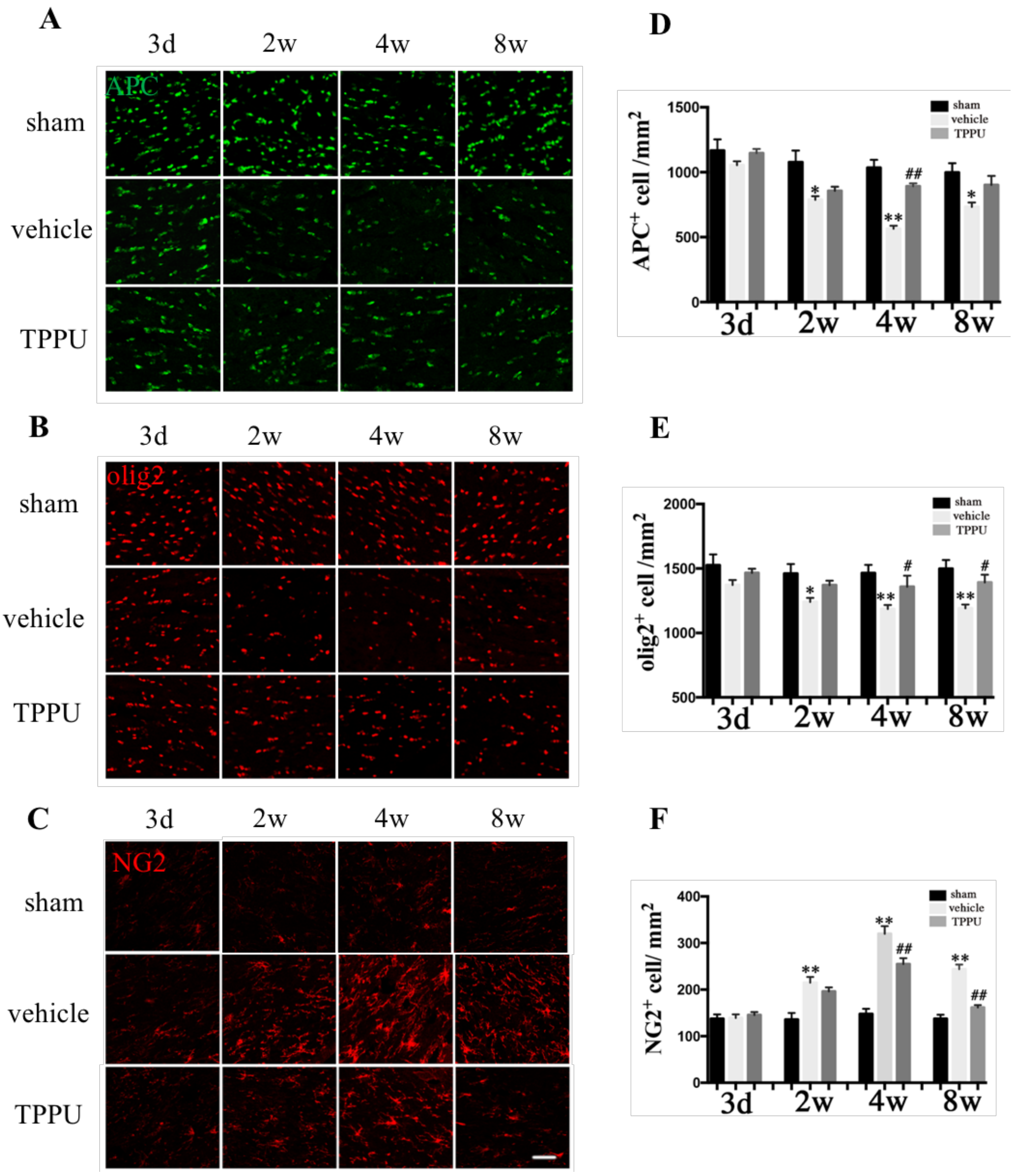

Supplementary Figure S4. TPPU enhances Oligodendrogenesis at different time points after bilateral carotid artery stenosis. **(A-C)** Representative immunofluorescent staining of APC (marker of mature oligodendrocytes, green), olig2 (marker of the oligodendrocyte lineage, red), and NG2 (marker of oligodendrocyte progenitor cell, red) in the corpus callosum (CC) of mice in sham, vehicle and TPPU treatment groups from days 3 to weeks 8 after operation. Scale bar represents 50 μm. (**D-F**) Statistical analysis of immunofluorescent staining of APC, olig2 and NG2 in the CC of sham, vehicle and

TPPU treatment groups, expressed as cells/mm^2. Values are expressed as mean ± SEM (n=5/group). *P<0.05, **P<0.01, vs sham group. $^{\#}P$<0.05, $^{\#\#}P$<0.01, vs vehicle group.

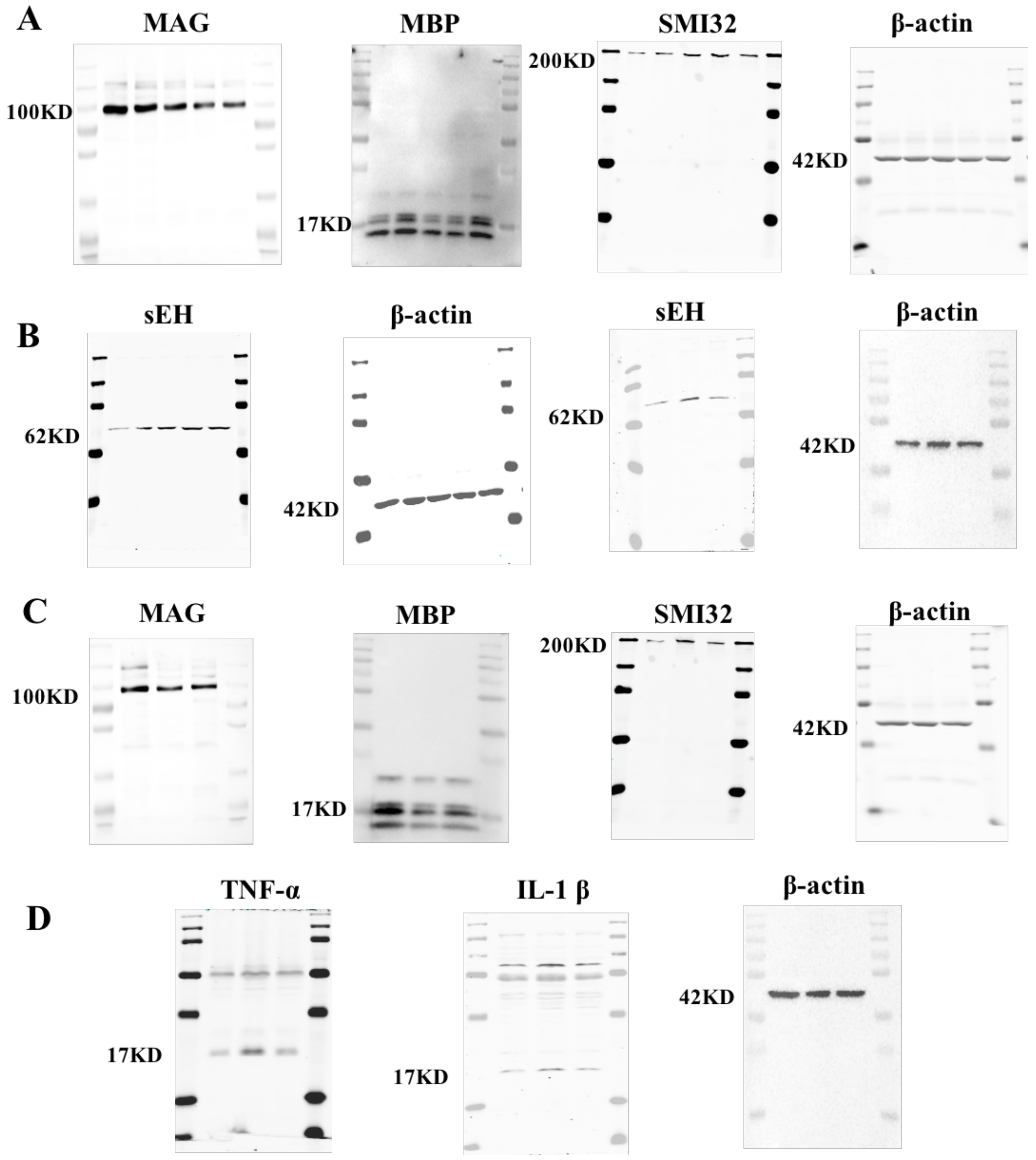

Supplementary Figure. The full images containing the whole blot with molecular weight markers. **(A)** The full images containing the whole blot with molecular weight markers in Fig.1. **(B)** The full images containing the whole blot with molecular weight markers in Fig.2. **(C)** The full images containing the whole blot with molecular weight markers in Fig.3. **(D)** The full images containing the whole blot with molecular weight markers in Fig.5.
